# Large Language Models in Multidisciplinary Decision-Making for Hepatopancreatobiliary Oncology: Retrospective Comparative Feasibility Study

**DOI:** 10.2196/101137

**Published:** 2026-09-17

**Authors:** Sung Jun Jo, Eui Hyuk Chong, Incheon Kang, Seok Jeong Yang, Beodeul Kang, Jung Sun Kim, Hong Jae Chon, Chang-il Kwon, Min Je Sung, Suk Pyo Shin, Chansik An, Ho Yeong Lim, Jeong-Sik Yu, Sujin Jang, Jung Ho Im, Kwang Hyun Ko, Sung Hwan Lee

**Affiliations:** 1 Department of Surgery, CHA University Bundang Medical Center, 59 Yatap-ro, Bundang-gu, Seongnam, Gyeonggi-do, Republic of Korea, 82 031-780-5000; 2 Department of Medical Oncology, CHA University Bundang Medical Center, Seongnam, Gyeonggi-do, Republic of Korea; 3 Department of Gastroenterology, CHA University Bundang Medical Center, Seongnam, Gyeonggi-do, Republic of Korea; 4 Department of Radiology, CHA University Bundang Medical Center, Seongnam, Gyeonggi-do, Republic of Korea; 5 Department of Nuclear Medicine, CHA University Bundang Medical Center, Seongnam, Gyeonggi-do, Republic of Korea; 6 Department of Radiation Oncology, CHA University Bundang Medical Center, Seongnam, Gyeonggi-do, Republic of Korea

**Keywords:** large language models, AI, natural language processing, clinical decision support, neoplasms, shared decision-making

## Abstract

**Background:**

Hepatopancreatobiliary (HPB) malignancies require complex treatment planning that often relies on multidisciplinary team (MDT) discussions. Large language models (LLMs) have recently been explored for clinical decision support, but their performance within real-world multidisciplinary decision environments remains unclear. In particular, the stability of LLM-generated recommendations—that is, whether a model produces the same answer when given the same clinical input—has rarely been examined.

**Objective:**

This study aimed to evaluate the stability of treatment recommendations generated by contemporary LLMs when identical HPB cases are queried repeatedly, and their concordance with the treatment decisions reached at an institutional MDT conference.

**Methods:**

This retrospective study included consecutive cases discussed at a single-center HPB MDT conference between September 1, 2024, and August 31, 2025. Standardized clinical case summaries derived from preconference documentation were provided to 4 LLMs (GPT-4o, GPT-5.2, Gemini 3 Pro, and Claude Sonnet 4.5) through their consumer web interfaces. Each model recommended a treatment among predefined MDT treatment options, and identical queries were repeated 4 times in separate sessions. Stability was quantified as the discordance rate relative to the initial response and, without privileging any single query, as the mean pairwise agreement and Fleiss κ across the 4 iterations. Concordance with MDT decisions was assessed using both the initial and modal responses, together with Cohen κ and class-wise *F*_1_-scores.

**Results:**

A total of 107 MDT cases were analyzed. Stability differed significantly across models (*P*=.01). Gemini 3 Pro showed the lowest discordance rate (mean 12.8%, SD 2.3%) and the highest reference-free agreement (Fleiss κ=0.737), whereas GPT-4o showed the highest discordance rate (mean 30.2%, SD 6.5%) and the lowest agreement (Fleiss κ=0.430). Concordance with MDT decisions ranged from 48.6% to 72.9% using the initial response and from 66.3% to 74.5% using the modal response, and the highest-performing model differed between the 2 definitions. Class-wise *F*_1_-score was consistently lower for surgery (0.400‐0.520) than for chemotherapy (0.621‐0.836). Complete discordance occurred in 17 of 107 (15.9%) cases and in none of the 31 anatomically unresectable cases (Fisher exact test, *P*=.003). Recurrent or on-treatment disease (adjusted odds ratio [OR] 5.40, 95% CI 1.65-17.68; *P*=.005), pancreatic tumor location (adjusted OR 7.37, 95% CI 2.03-26.78; *P*=.002), and low MDT agreement level (adjusted OR 10.33, 95% CI 1.54-69.38; *P*=.016) were independently associated with complete discordance.

**Conclusions:**

LLM-generated treatment recommendations demonstrated moderate alignment with MDT decisions in HPB oncology. Importantly, response stability varied substantially across models, indicating that concordance alone is insufficient for evaluating LLMs as clinical decision support tools. These findings suggest that LLMs may serve as a reasoning-support layer in MDT-like decision environments, but their response stability must be systematically characterized before clinical integration.

## Introduction

Hepatopancreatobiliary (HPB) malignancies represent some of the most complex diseases in oncology, often requiring careful integration of diagnostic imaging, pathology, surgical evaluation, and systemic therapy planning [1-3]. Optimal management frequently depends on multidisciplinary decision-making involving hepatobiliary surgeons, gastroenterologists, medical oncologists, radiologists, and radiation oncologists [1,2,4]. Multidisciplinary team (MDT) discussions have, therefore, become an essential component of modern HPB cancer care [1,4-6]. Previous studies have shown that MDT-based management can improve staging accuracy, optimize treatment selection, and enhance adherence to evidence-based guidelines [5-7]. However, MDT discussions are inherently resource-intensive and require coordinated input from multiple specialists [1,4,8]. As the complexity and number of oncologic cases increase, maintaining efficient and consistent MDT decision-making can be challenging, particularly in settings with limited expert availability [5,8].

Recent advances in AI, particularly large language models (LLMs), have introduced new possibilities for supporting complex clinical decision processes [9-11]. Unlike traditional rule-based decision support systems, LLMs are capable of integrating heterogeneous information and generating structured reasoning in natural language [9-11]. In emerging agentic AI frameworks, LLMs are increasingly considered a central reasoning layer capable of orchestrating multiple information sources and decision pathways [9,12]. In such systems, the role of LLMs is not merely to provide isolated recommendations but to coordinate complex reasoning processes and integrate diverse inputs—functions that conceptually resemble the orchestration process occurring in multidisciplinary clinical discussions [1,9,12].

Despite the rapidly growing interest in applying LLMs to clinical decision support, most existing studies have primarily evaluated the accuracy or concordance of LLM-generated recommendations compared with physician decisions or clinical guidelines [10-13]. Although these studies provide important insights into the decision-making capabilities of LLMs, they do not fully address whether LLMs can function within complex clinical decision-making environments, such as MDT discussions [1,11,13,14]. In particular, little is known about the stability of LLM-generated recommendations when presented with identical clinical information, or about the degree to which such recommendations align with real-world MDT decisions [11,13,14].

Evidence from adjacent applications of LLMs to clinical text frames what can reasonably be expected in this setting. Validation studies of information extraction from oncology records report accuracy that is high but variable across document types and terminology [15], establishing that these models can parse clinical narratives while leaving open whether the judgment built on that parsing is sound. The experience of deployed clinical prediction models is instructive in this regard: a widely implemented proprietary sepsis prediction model performed substantially worse on external validation at a single center than its development data had suggested [16,17], indicating that performance must be established in the institution where a tool would be used rather than assumed from reported figures. Tumor board concordance studies in breast and primary liver cancer have begun this work [18,19], but most rely on curated case profiles or a single query per case, so response stability and institution-level validity remain largely unexamined. Reporting standards for this class of study have only recently been formalized [20]. Therefore, this study aimed to investigate the feasibility of LLMs as a potential orchestration component in multidisciplinary oncologic decision-making. Using real-world cases discussed in an HPB MDT conference, we conducted a comparative evaluation across 4 contemporary LLMs.

## Methods

### Study Design and Population

This retrospective observational study evaluated the feasibility of LLMs as a potential orchestration component in multidisciplinary decision-making for HPB malignancies. All consecutive cases discussed at the HPB MDT conference at our institution between September 1, 2024, and August 31, 2025, were screened for eligibility. The MDT conference included specialists from hepatobiliary surgery, gastroenterology, medical oncology, radiology, and radiation oncology, and treatment decisions were determined through multidisciplinary discussion.

Cases were eligible for inclusion if they involved patients with HPB malignancies undergoing MDT discussion for treatment planning. Cases were excluded if they met any of the following criteria: (1) cases discussed solely for biliary drainage without a definitive oncologic diagnosis, (2) patients with synchronous or metachronous double primary malignancies, or (3) metastases to the HPB system from a non-HPB primary tumor. The final study cohort consisted of cases meeting the eligibility criteria during the study period.

### Clinical Variables and Definitions

Clinical variables used in the analysis were extracted from MDT records and electronic medical records. Age and sex were obtained from patient demographic data. Tumor markers, including carcinoembryonic antigen and carbohydrate antigen 19‐9, were recorded based on the laboratory results available at the time of the MDT discussion. Disease status was classified as primary disease, recurrent disease, or on-treatment disease. Tumor location was categorized as biliary tract, pancreas, or ampulla of Vater based on the primary tumor origin. Biliary tract cancers included intrahepatic cholangiocarcinoma, perihilar cholangiocarcinoma, distal common bile duct cancer, and gallbladder cancer. For pancreatic cancer cases, tumor location was further classified as head, neck, body, tail, or diffuse involvement according to radiologic reports. The main referring department was categorized as gastroenterology, medical oncology, or surgical oncology. The MDT agreement level was recorded based on the documented MDT conference conclusions and categorized as high confidence, moderate confidence, or low confidence, reflecting the degree of consensus among participating specialists. Caregiver support was determined based on the family members documented as accompanying the patient to the MDT conference, together with the patient’s recorded residential address. Support was classified as present when a spouse attended. When the accompanying relative was an adult child or other family member, support was classified as present unless the patient resided in a rural area distant from that relative, in which case it was classified as absent. Cases with no accompanying family member were classified as absent.

Treatment recommendations were categorized as surgery, chemotherapy, radiation therapy, active surveillance, or further evaluation. Sequential and combined plans were assigned to the modality initiated first, so that a plan of neoadjuvant chemotherapy followed by reassessment for resection was categorized as chemotherapy. The same rules were applied to the documented MDT conclusion and to the model output. Full mapping rules and the institutional protocols in effect during the study period are provided in Multimedia Appendix 1.

Anatomic resectability was assessed retrospectively for all cases by 3 hepatobiliary and pancreatic surgeons reading together in a single consensus session. Assessors reviewed the cross-sectional imaging and classified each case as unresectable, borderline resectable, or resectable on the basis of the anatomic relationship between the tumor and the vasculature, with disagreements resolved by discussion until a single classification was agreed upon. Assessors were blinded to both the MDT decision and all model output. Because classification was reached by consensus rather than by independent reading, interrater agreement could not be calculated.

### Clinical Information Used as Model Input

Clinical information presented during the MDT conference was compiled and used as input for the LLMs. The case summaries were derived from the referral and preconference documentation prepared when the patient entered the MDT workflow, before the conference and before any treatment decision was made. For this study, the documentation was reformatted into a standardized template without alteration of clinical content. The documented MDT conclusion was not included in the model input, and only information available at the time of the conference was used. The input data included patient demographics (age and sex), radiologic findings from abdominal computed tomography or magnetic resonance imaging and chest computed tomography, histopathologic results from biopsy, when available, positron emission tomography findings, and laboratory data, including total bilirubin, aspartate aminotransferase, alanine aminotransferase, and tumor markers such as carbohydrate antigen 19‐9 and carcinoembryonic antigen. Radiologic and pathologic information was summarized in text format based on formal clinical reports rather than raw imaging or laboratory datasets. All clinical information was organized into a standardized textual case summary before being provided to the models.

### AI Models and Query Framework

Four contemporary LLMs were evaluated in this study: GPT-4o, GPT-5.2, Gemini 3 Pro, and Claude Sonnet 4.5. All models were accessed using their publicly available versions without additional fine-tuning. Each model received identical clinical case summaries and was asked to recommend the most appropriate treatment strategy among predefined options reflecting common MDT decisions: surgery, chemotherapy, radiation therapy, active surveillance, or further evaluation. The models were instructed to provide a clear recommendation with a brief explanation.

All queries were performed through the consumer web interfaces (ChatGPT, the Gemini app, and claude.ai) rather than provider APIs. Models were queried in separate blocks between November 2025 and May 2026: GPT-4o in November-December 2025, GPT-5.2 in December 2025-January 2026, Gemini 3 Pro in February 2026, and Claude Sonnet 4.5 over approximately 1 month thereafter. Query blocks were bounded by the periods during which each model was selectable in its consumer interface; exact query dates were not logged prospectively. The model was selected explicitly in the model picker for every query rather than relying on the application default.

GPT-5.2 was used in the default interface configuration in which the provider’s router determines whether a given query is served by the Instant or the Thinking variant; this routing is not visible or user-configurable, and neither Thinking nor Pro was manually selected. More generally, these interfaces do not expose decoding parameters, so temperature, top-p, and system-level instructions remained at provider defaults. Browsing and retrieval were disabled, and account-level memory and personalization were turned off for all providers. Each of the 4 repeated queries for a given case was submitted in a new session, and each session was deleted after the response was recorded, so that no query and no case could enter the context of a subsequent one. Exact query dates were, therefore, not retained in the interface history and are reported at the level of the query block.

To evaluate response stability, identical queries were submitted to each model 4 times using the same clinical input and prompt. LLMs generate responses through probabilistic processes, which may produce variable outputs even when identical inputs are provided. Repeated queries were, therefore, used to assess the consistency of treatment recommendations under identical clinical conditions.

### Qualitative Review of Model Rationales

In addition to the categorical recommendation, the free-text rationale accompanying each response was retained. In a post hoc analysis, 4 cases were purposively selected for detailed review: 2 discordant cases representing opposite directions of error across the surgical decision boundary and 2 concordant cases as comparators. Selection was purposive rather than random, and no quantitative inference was drawn from these cases. Each rationale was reviewed against the corresponding MDT record by the study investigators. The annotated cases are presented in Multimedia Appendix 2.

### Study Outcomes

The primary outcomes of this study were the stability of LLM-generated treatment recommendations and their clinical concordance with MDT decisions. Stability was defined as the consistency of treatment recommendations generated by each LLM when identical clinical information was provided repeatedly. To assess this, identical queries were submitted 4 times to each model using the same clinical case summary. Concordance was defined as agreement between the treatment recommendation generated by the LLM and the final treatment decision determined during the MDT conference. Concordance analysis was performed using both the first response and the modal response across the 4 repeated queries. The secondary outcome was to identify clinical factors associated with complete discordance, defined as cases in which all evaluated LLM models generated treatment recommendations that differed from the MDT decision.

### Statistical Analysis

Continuous variables are presented as mean (SD) or median (IQR), as appropriate, and categorical variables are presented as numbers and percentages. Comparisons between groups were performed using the 2-tailed Student *t* test or Mann-Whitney *U* test for continuous variables and the chi-square test or Fisher exact test for categorical variables, as appropriate. To evaluate the stability of LLM-generated treatment recommendations, discordance rates were calculated by comparing treatment categories from repeated queries (queries 2‐4) with the initial response (query 1) for each model. Because this definition designates the initial response as the reference, agreement across the 4 queries was additionally quantified without privileging any single query, using mean pairwise agreement across all 6 query pairs and Fleiss κ, treating the 4 iterations as raters. Bootstrap 95% CIs for Fleiss κ were obtained from 4000 resamples. Concordance with MDT decisions was initially assessed using the first response of each model. However, using a single response as the reference may misrepresent a model whose first output is atypical, since a recommendation that differs from the first response is counted as discordant, even when it is the recommendation the model produces most often. Concordance was, therefore, also assessed using the modal response, defined as the treatment category most frequently recommended across the 4 repeated queries; cases without a unique mode were excluded (n=6, 10, 5, and 9 for GPT-4o, GPT-5.2, Gemini 3 Pro, and Claude Sonnet 4.5, respectively). Under each definition, overall concordance, Cohen κ, and class-wise *F*_1_-scores were calculated against the MDT decision. Class-wise *F*_1_-score was calculated only for treatment categories represented by at least 5 MDT decisions, since precision and recall are unstable below this frequency; radiation therapy (n=1), active surveillance (n=2), and further evaluation (n=2) fell below this threshold, so *F*_1_-score is reported for surgery (n=25) and chemotherapy (n=77) only. Discordance rates among models were compared using the chi-square test. To identify clinical factors associated with complete discordance, univariate logistic regression analysis was performed. Variables for the multivariate model were prespecified on clinical grounds rather than selected by univariate significance and were limited to 3 given 17 events; Firth’s penalized maximum likelihood was used because of the small number of events. Odds ratios (ORs) with 95% CIs were calculated. All statistical analyses were performed using R software (The R Core Team). *P*<.05 (2-sided) was considered statistically significant.

### Ethical Considerations

The study protocol conformed to the ethical guidelines of the Declaration of Helsinki and was approved by the institutional review board of Bundang CHA Medical Center (IRB 2026-03-057). The requirement for informed consent was waived owing to the retrospective nature of the study. All patient data were deidentified before analysis.

## Results

### Patient Characteristics

**Figure 1.**
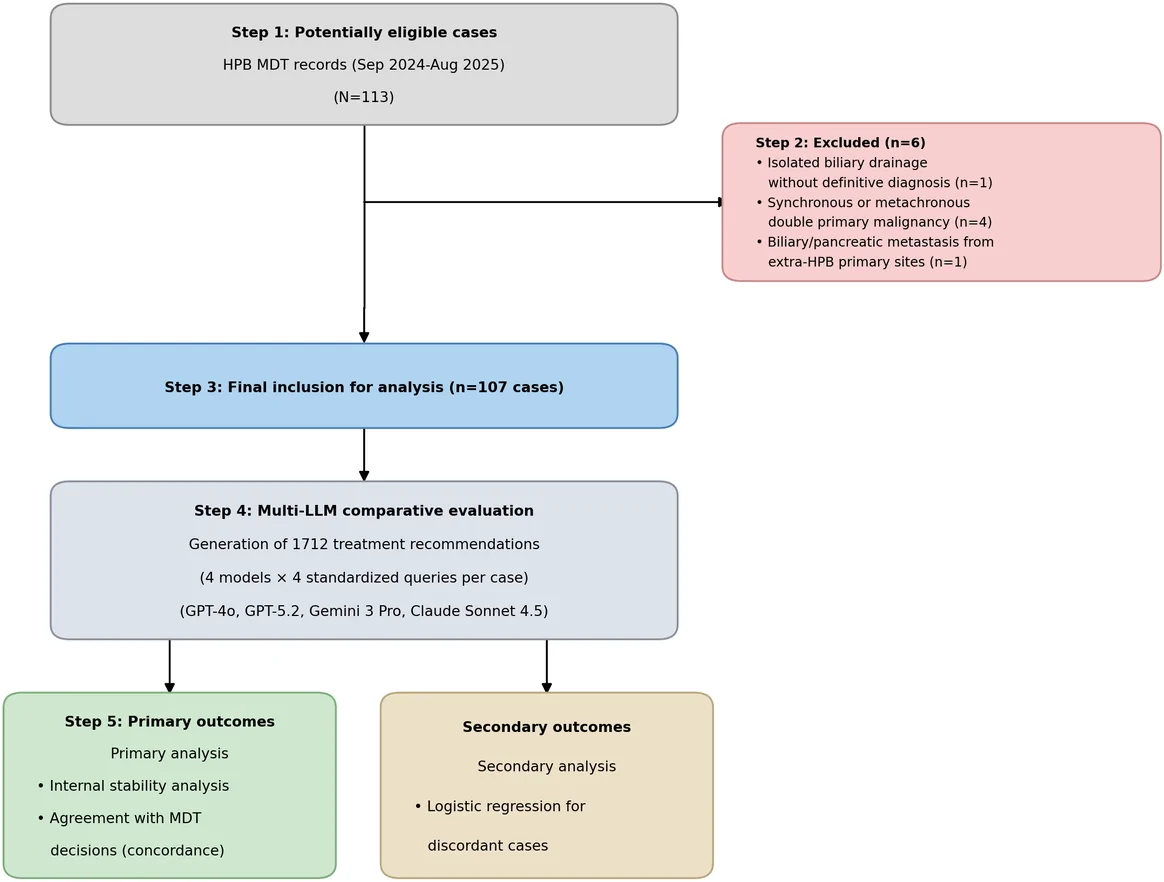
Flow diagram of patient selection. HPB: hepatopancreatobiliary; LLM: large language model; MDT: multidisciplinary team.

The flow diagram of patient inclusion is shown in Figure 1. Between September 1, 2024, and August 31, 2025, a total of 113 cases were discussed at the HPB MDT conference and were screened for eligibility. After applying the predefined exclusion criteria—including cases discussed solely for biliary drainage, patients with double primary malignancies, and metastases from non-HPB primary tumors—107 cases were included in the final analysis.

**Table 1.** Characteristics of patients.

Variables	Primary (n=77)	Recurrent or on-treatment (n=30)	*P* value
Age (y), mean (SD)	66.4 (9.6)	65.0 (11.0)	.66
Sex, n (%)			.66
Male	46 (59.7)	20 (66.7)	
Female	31 (40.3)	10 (33.3)	
Main department, n (%)			<.001
Gastroenterology	59 (76.6)	4 (13.3)	
Medical oncology	3 (3.9)	23 (76.7)	<.001
Surgical oncology	15 (19.5)	3 (10.0)	
Tumor location (organ), n (%)			>.99
Biliary tract	51 (66.2)	20 (66.7)	
Pancreas	24 (31.2)	9 (30.0)	
Ampulla of Vater	2 (2.6)	1 (3.3)	
Bile duct cancer type, n (%)^a^			.07
Intrahepatic	12 (23.5)	5 (25)	
Hilar (perihilar)	30 (58.8)	6 (30)	
Distal common bile duct	5 (9.8)	5 (25)	
Gallbladder	4 (7.8)	4 (20)	
Pancreatic cancer site, n (%)^a^			.52
Head	16 (66.7)	4 (44.4)	
Neck	1 (4.2)	0 (0)	
Body	3 (12.5)	3 (33.3)	
Tail	3 (12.5)	1 (11.1)	
Diffuse	1 (4.2)	1 (11.1)	
Anatomic resectability, n (%)			.28
Unresectable	20 (26.0)	11 (36.7)	
Borderline resectable	33 (42.9)	8 (26.7)	
Resectable	24 (31.2)	11 (36.7)	
MDT^b^ decision, n (%)			<.001
Surgery	12 (15.6)	13 (43.3)	
Chemotherapy	64 (83.1)	13 (43.3)	
Radiation therapy	0 (0)	1 (3.3)	
Active surveillance	0 (0)	2 (6.7)	
Further evaluation	1 (1.3)	1 (3.3)	
MDT agreement level, n (%)			.32
High confidence	66 (85.7)	22 (73.3)	
Moderate confidence	7 (9.1)	5 (16.7)	
Low confidence	4 (5.2)	3 (10.0)	
Tumor marker			
CEA^c^ (ng/mL), median (IQR)	2.81 (2.21‐4.33)	2.75 (1.96‐3.93)	.71
CA 19‐9^d^ (U/mL), median (IQR)	143.5 (45.0‐400.0)	37.8 (15.2‐267.8)	.03
Caregiver support, n (%)			>.99
Present	72 (93.5)	28 (93.3)	
Absent	5 (6.5)	2 (6.7)	

a Percentages for bile duct cancer type are calculated among patients with biliary tract cancer (n=51 in the primary group and n=20 in the recurrent or on-treatment group), and percentages for pancreatic cancer site among patients with pancreatic cancer (n=24 and n=9, respectively). All other percentages are calculated within the full column.

b MDT: multidisciplinary team.

c CEA: carcinoembryonic antigen.

d CA 19-9: carbohydrate antigen 19-9.

The baseline characteristics of the study population, stratified by disease status, are summarized in Table 1. Among the 107 included cases, 77 (72.0%) had primary disease, whereas 30 (28.0%) had recurrent or on-treatment disease.

The mean age was 66.4 (SD 9.6) years in the primary disease group and 65.0 (SD 11.0) years in the recurrent or on-treatment group (*P*=.66), and the proportion of male patients was similar between groups (46/77, 59.7% vs 20/30, 66.7%; *P*=.66). Regarding tumor location, the distribution of biliary tract, pancreas, and ampulla of Vater tumors was comparable between groups (*P*>.99). However, the main referring department differed significantly, with gastroenterology accounting for the majority of primary disease cases (59/77, 76.6%), whereas medical oncology accounted for most recurrent or on-treatment cases (23/30, 76.7%; *P*<.001). In terms of MDT treatment decisions, chemotherapy was the predominant recommendation in the primary disease group (64/77, 83.1%), whereas surgery and chemotherapy were equally recommended (13/30, 43.3% each) in the recurrent or on-treatment group (*P*<.001). The distribution of MDT agreement levels and most tumor markers were comparable between groups, although CA 19‐9 levels were higher in the primary disease group (primary: median 143.5, IQR 45.0‐400.0 vs recurrent or on-treatment: median 37.8, IQR 15.2‐267.8; *P*=.03).

### Stability of LLM Responses

The stability of LLM-generated treatment recommendations was assessed by calculating discordance rates across repeated queries. For each model, treatment recommendations from repeated queries (queries 2‐4) were compared with the initial response (query 1). The mean discordance rates differed among the evaluated models. Gemini 3 Pro demonstrated the lowest discordance rate (mean 12.8%, SD 2.3%), followed by GPT-5.2 (mean 22.7%, SD 5.8%), Claude Sonnet 4.5 (mean 28.0%, SD 1.5%), and GPT-4o (mean 30.2%, SD 6.5%). The difference in discordance rates among models was statistically significant (*P*=.01). Detailed discordance rates for each repeated query are presented in Table 2.

**Table 2.** Response stability and concordance of large language models (LLMs) with multidisciplinary team (MDT) decisions for hepatopancreatobiliary (HPB) treatment recommendations^a^.

Model	Discordance vs initial response (n=107)				Reference-free agreement		Concordance with MDT decision		
	#2, n (%)	#3, n (%)	#4, n (%)	Mean (SD)	Pairwise agreement (%)	Fleiss κ (95% CI)	Concordance (n=107), n (%)	*F*_1_-score for surgery	*F*_1_-score for chemotherapy
GPT-4o	26 (24.3)	42 (39.3)	29 (27.1)	30.2 (6.5)	67.8	0.430 (0.33‐0.52)	78 (72.9)	0.520	0.836
GPT-5.2	21 (19.6)	33 (30.8)	19 (17.8)	22.7 (5.8)	74.5	0.535 (0.43‐0.63)	73 (68.2)	0.510	0.803
Gemini 3 Pro	11 (10.3)	13 (12.1)	17 (15.9)	12.8 (2.3)	87.1	0.737 (0.64‐0.82)	72 (67.3)	0.444	0.770
Claude Sonnet 4.5	30 (28.0)	28 (26.2)	32 (29.9)	28.0 (1.5)	74.0	0.572 (0.48‐0.66)	52 (48.6)	0.400	0.621

a Discordance rates were calculated by comparing the treatment category from each repeated query (#2 to #4) with the initial response (#1), and differed significantly across the 4 models (chi-square test, *P*=.01). Pairwise agreement is the mean agreement across all 6 query pairs, and Fleiss κ treats the 4 iterations as raters. Concordance and *F*_1_-scores are based on the initial response, with the MDT decision as the reference standard. *F*_1_-score is reported for surgery and chemotherapy only.

Because these discordance rates used the initial response as the reference, agreement across the 4 queries was additionally quantified without designating any single query as the reference. Mean pairwise agreement across all 6 query pairs and Fleiss κ, treating the 4 iterations as raters, were 67.8% and 0.430 for GPT-4o, 74.5% and 0.535 for GPT-5.2, 87.1% and 0.737 for Gemini 3 Pro, and 74.0% and 0.572 for Claude Sonnet 4.5. Gemini 3 Pro was the most stable model under every measure. The ordering of the remaining models was not preserved: Claude Sonnet 4.5 showed a higher discordance rate than GPT-5.2 (mean 28.0%, SD 1.5% vs mean 22.7%, SD 5.8%) but also higher reference-free agreement (Fleiss κ 0.572 vs 0.535).

### Concordance With MDT Decisions

**Figure 2.**
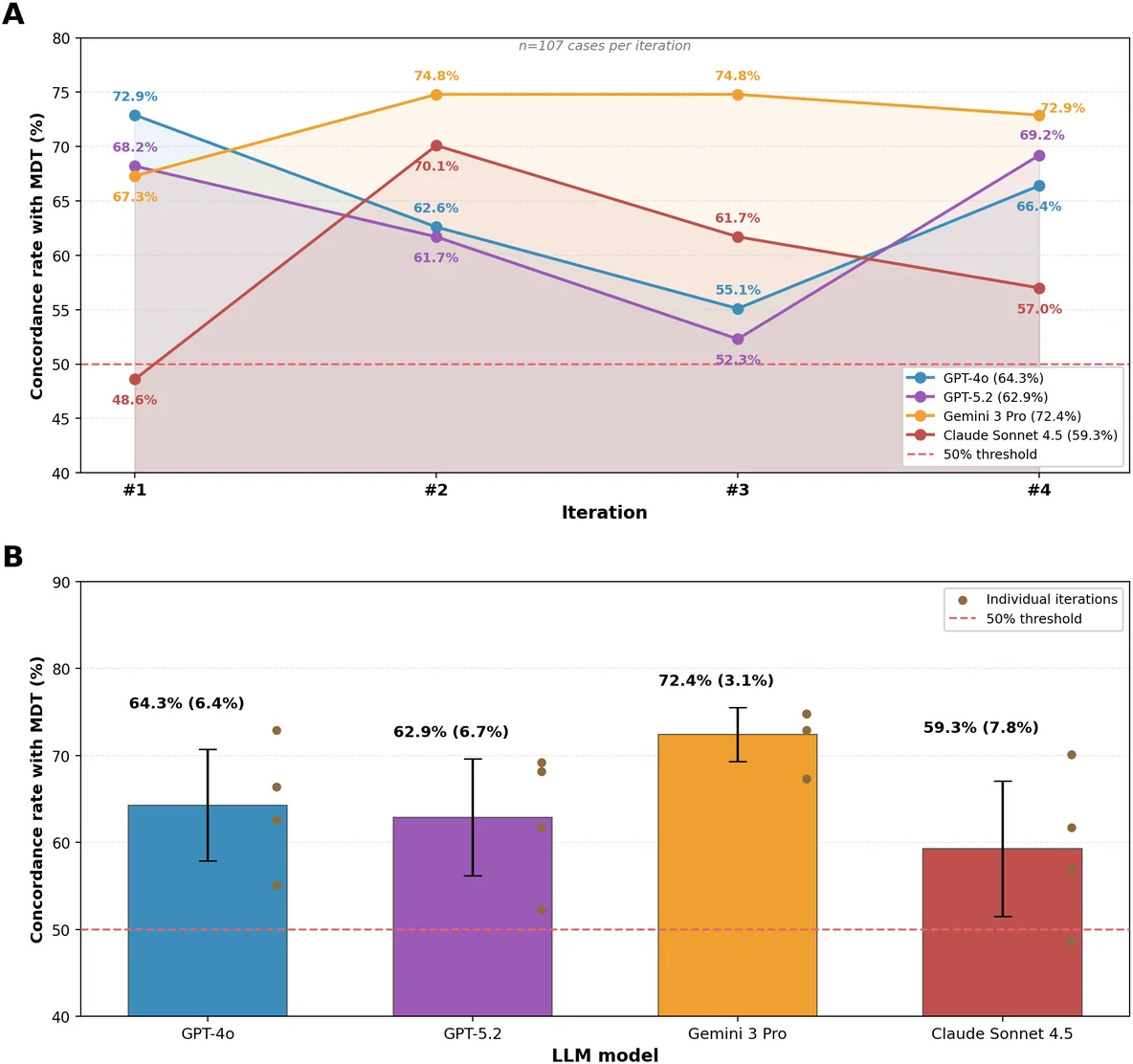
Concordance between large language model (LLM) recommendations and multidisciplinary team (MDT) decisions. (A) Concordance rates between LLM-generated treatment recommendations and MDT decisions across 4 repeated queries for each model. (B) Mean concordance rates averaged across the 4 iterations for each model. Error bars represent SD.

The concordance between LLM-generated treatment recommendations and MDT decisions across repeated queries is shown in Figure 2. In the first iteration (consisting of 107 cases), concordance rates were 72.9% (n=78) for GPT-4o, 68.2% (n=73) for GPT-5.2, 67.3% (n=72) for Gemini 3 Pro, and 48.6% (n=52) for Claude Sonnet 4.5. Across repeated queries, Gemini 3 Pro showed consistently high concordance rates (67.3%, 74.8%, 74.8%, and 72.9%), whereas GPT-4o, GPT-5.2, and Claude Sonnet 4.5 showed greater variability across iterations. When averaged across all 4 iterations, the mean concordance rates were 72.4% (SD 3.1%) for Gemini 3 Pro, 64.3% (SD 6.4%) for GPT-4o, 62.9% (SD 6.7%) for GPT-5.2, and 59.3% (SD 7.8%) for Claude Sonnet 4.5.

Class-wise performance based on the first response is shown in Table 2. Across all models, *F*_1_-scores were consistently higher for chemotherapy (0.621‐0.836) than for surgery (0.400‐0.520), indicating that the models identified candidates for systemic therapy more reliably than candidates for surgery.

### Concordance Using the First Response vs the Modal Response

Because concordance was assessed using the first response of each model, an additional analysis was performed using the modal response across the 4 repeated queries (Table 3). The ranking of models differed between the 2 reference responses. Using the first response, GPT-4o showed the highest concordance (78/107, 72.9%) and Claude Sonnet 4.5 the lowest (52/107, 48.6%), whereas, using the modal response, Gemini 3 Pro showed the highest concordance (76/102, 74.5%). The difference between the 2 reference responses was largest for Claude Sonnet 4.5, in which concordance increased from 52 of 107 (48.6%) to 65 of 98 (66.3%) and Cohen κ from 0.115 to 0.356, and it was smallest for GPT-5.2 (73/107, 68.2% and 66/97, 68.0%).

**Table 3.** Concordance with multidisciplinary team (MDT) decisions using the first response vs the modal response.

Model	Concordance		Cohen κ	
	First response, n/N (%)	Modal response, n/N (%)	First response	Modal response
GPT-4o	78/107 (72.9)	70/101 (69.3)	0.434	0.335
GPT-5.2	73/107 (68.2)	66/97 (68.0)	0.369	0.306
Gemini 3 Pro	72/107 (67.3)	76/102 (74.5)	0.286	0.434
Claude Sonnet 4.5	52/107 (48.6)	65/98 (66.3)	0.115	0.356

### Predictors of Complete Discordance

Among the included cases, complete discordance—defined as cases in which all evaluated LLMs generated treatment recommendations different from the MDT decision—was observed in 17 of 107 (15.9%) cases. Complete discordance did not occur in any of the 31 unresectable cases, compared with 9 of 41 borderline resectable and 8 of 35 resectable cases (Fisher exact test, *P*=.003); because no events occurred in the unresectable stratum, resectability could not be entered into the regression model. To identify clinical factors associated with complete discordance, logistic regression analyses were performed (Table 4). In the univariate analysis, recurrent or on-treatment disease (OR 5.00, 95% CI 1.69‐14.82; *P*=.004), pancreatic tumor location (OR 3.98, 95% CI 1.35‐11.67; *P*=.01), and low MDT agreement level (OR 5.25, 95% CI 1.03‐26.66; *P*=.045) were significantly associated with complete discordance. In the multivariate analysis, recurrent or on-treatment disease (adjusted OR 5.40, 95% CI 1.65‐17.68; *P*=.005), pancreatic tumor location (adjusted OR 7.37, 95% CI 2.03‐26.78; *P*=.002), and low MDT agreement level (adjusted OR 10.33, 95% CI 1.54‐69.38; *P*=.02) remained independent predictors of complete discordance.

**Table 4.** Univariate and multivariate analyses of predictors of complete discordance.

Variables	Crude OR^a^ (95% CI)	*P* value	Adjusted OR (95% CI)	*P* value
Sex (male vs female)	1.17 (0.40-3.44)	.77	—^b^	
Age (per year)	0.98 (0.95-1.00)	.49	—	
Disease status: recurrent vs primary	5.00 (1.69-14.82)	.004	5.40 (1.65-17.68)	.005
Tumor location: pancreas vs biliary	3.98 (1.35-11.67)	.01	7.37 (2.03-26.78)	.002
Main department (reference: gastroenterology)				
Medical oncology	4.24 (1.37-13.07)	.01	—	
Surgical oncology	0.47 (0.05-4.10)	.50	—	
MDT^c^ agreement (reference: high)				
Moderate	2.33 (0.55-9.96)	.25	—	
Low	5.25 (1.03-26.66)	.045	10.33 (1.54-69.38)	.02
CEA^d^ (per ng/mL)	1.00 (1.00-1.00)	.93	—	
CA 19‐9^e^ (per U/mL)	1.00 (1.00-1.00)	.66	—	

a OR: odds ratio.

b Not available.

c MDT: multidisciplinary team.

d CEA: carcinoembryonic antigen.

e CA 19‐9: carbohydrate antigen 19-9.

## Discussion

### Principal Findings

HPB malignancies require complex treatment planning that integrates radiologic findings, pathology, surgical considerations, and systemic therapy strategies [1-3]. Consequently, MDT discussions have become a cornerstone of modern oncologic care, with previous studies demonstrating that MDT-based management can improve diagnostic accuracy, treatment selection, and, in some settings, patient survival outcomes [1-3,5-7,21]. However, MDT decision-making also requires substantial coordination of heterogeneous clinical information and expert perspectives, making the process resource-intensive and difficult to scale across health care systems [4,7,22].

Recent advances in LLMs have raised interest in their potential role in supporting complex clinical reasoning [9-13]. Most prior studies, however, have primarily evaluated the accuracy or guideline concordance of LLM-generated recommendations rather than examining how these systems behave within real-world multidisciplinary decision environments [11-14]. In this study, using real-world HPB MDT cases, we evaluated the feasibility of LLMs operating within an MDT-like clinical decision context [1,8,11,14]. Our results show that, while LLM-generated recommendations demonstrated moderate alignment with MDT decisions, their outputs exhibited model-dependent variability and diverged most frequently in clinically complex scenarios such as pancreatic malignancies and cases with low MDT consensus.

An important observation from this study relates to the stability of LLM-generated treatment recommendations. In clinical decision-making, the consistency of recommendations is essential because clinicians expect similar conclusions when the same clinical information is presented. However, LLMs generate responses through probabilistic processes, which may lead to variability even when identical inputs are provided. Despite the increasing interest in applying LLMs to clinical decision support, the stability of model-generated recommendations in real-world clinical scenarios has rarely been examined. By repeatedly querying multiple LLMs with identical MDT case summaries, our study demonstrates that stability varies substantially across models. These findings highlight that response stability represents an important consideration when integrating LLM-based systems into clinical workflows and suggest that some models may be more suitable than others for supporting complex multidisciplinary decision-making.

### Concordance and Its Interpretation

In our study, we observed a moderate level of concordance between LLM-generated treatment recommendations and MDT decisions, with overall agreement rates generally ranging between approximately 60% and 70% across models. Although MDT decisions are widely regarded as the current standard for complex oncologic decision-making, they do not necessarily represent a single definitive “correct” answer. Rather, MDT recommendations emerge from multidisciplinary discussions that integrate clinical expertise, institutional practice patterns, and patient-specific considerations. Consequently, some degree of variation in treatment recommendations can be expected even among expert clinicians.

Within this context, the concordance observed in our study suggests that LLM-generated recommendations may capture certain elements of multidisciplinary clinical reasoning. Discordant cases may, therefore, not necessarily represent incorrect recommendations but instead reflect institutional treatment practices, patient-specific considerations, or contextual factors that may not be fully captured in structured case summaries. Taken together, these findings indicate that LLM recommendations demonstrate a meaningful degree of alignment with MDT-based clinical decision-making.

Three observations qualify these concordance estimates. First, chemotherapy accounted for 77 of the 107 (72.0%) MDT decisions, so overall concordance is heavily influenced by the ability to identify this dominant category. Class-wise *F*_1_-scores were consistently lower for surgery than for chemotherapy across all models, indicating that the models were least reliable for the decision carrying the greatest clinical consequence. Second, concordance estimates depended on which of the repeated responses was used as the reference, and the ranking of models differed between the first response and modal response definitions. We therefore report both rather than designating one as correct: the first response reflects what a clinician would receive from a single query, whereas the modal response reflects the model’s central tendency, and the two answer different questions. That they diverge is itself a manifestation of response instability and supports our central point that concordance and stability should not be interpreted independently. Third, treatment recommendations were categorized by the modality initiated first, so a model recommendation of neoadjuvant chemotherapy followed by surgery and an MDT decision of upfront surgery were counted as discordant even though both anticipate resection; concordance as reported, therefore, reflects agreement on the immediate treatment modality rather than on the complete therapeutic strategy (Multimedia Appendix 1).

Two further considerations bear on how these estimates should be used. Concordance figures reported in other tumor board series, including approximately 80% for cholangiocarcinoma in a single-center liver MDT study [19], are not directly comparable to ours: that design used curated case summaries and a coarser outcome definition, whereas our outcome had 5 categories and our cases were consecutive real presentations, both of which lower concordance mechanically. More importantly, the rationales accompanying model recommendations were fluent and clinically framed, regardless of whether the recommendation was correct (Multimedia Appendix 2), consistent with evidence that generated reasoning does not reliably reflect the computation that produces the answer [23]. A clinician reviewing an LLM recommendation, therefore, cannot treat the persuasiveness of its justification as a quality signal, and verification requires checking each stated premise against the source reports rather than assessing the coherence of the explanation.

### Predictors of Discordance

Discordance between LLM-generated recommendations and MDT decisions in our study was not randomly distributed but occurred more frequently in specific clinical contexts, particularly in pancreatic tumors and cases with low MDT agreement. While the association with low MDT confidence is expected, the higher discordance observed in pancreatic malignancies warrants further consideration. Treatment planning for pancreatic cancer often requires nuanced interpretation of tumor resectability, vascular involvement, and patient-specific clinical factors, which can introduce variability in clinical decision-making.

Previous studies have also shown that substantial variation may exist among expert teams in the assessment of pancreatic cancer resectability and treatment strategies [1,24,25]. In multicenter evaluations of pancreatic cancer cases reviewed by multiple MDTs, notable differences in resectability assessments and treatment recommendations have been reported [24,25]. These findings suggest that pancreatic cancer represents a clinical scenario in which decision-making itself is inherently complex and subject to differing expert interpretations [1,24,25]. In this context, the discordance observed between LLM recommendations and MDT decisions in our study may reflect the intrinsic difficulty of these cases rather than a systematic limitation of model reasoning.

### Clinical Implications

Our findings highlight a potential and pragmatic role for LLMs in multidisciplinary clinical decision environments. Rather than representing a fully autonomous clinical decision system, LLM-based approaches may function as reasoning-support tools that assist clinicians in organizing complex clinical information and generating structured treatment considerations. In this sense, the use of LLMs within MDT-like contexts may represent a feasible direction for further investigation.

This study evaluated retrospective case summaries in a controlled setting. It did not assess safety, clinician interaction, workflow impact, or patient outcomes, and the implications discussed here are, therefore, framed as conditions that would have to be met before any clinical use, not as evidence that such use is warranted. Any such use would require safeguards consistent with the limitations observed here. Because model outputs varied across identical queries and were least accurate for surgical decisions, every recommendation would require review by the treating team, which retains clinical accountability. Deployment would also require version pinning with periodic revalidation, given that model behavior changes with each release, as well as the processing of only deidentified text.

At the same time, the application of AI to complex oncologic decision-making will likely require the integration of additional components beyond language-based reasoning. Comprehensive clinical decision systems would need to incorporate multimodal capabilities, including agents capable of directly interpreting imaging data, pathology findings, and other raw clinical inputs. Our findings indicate that the reasoning component of such systems, represented by LLM-based synthesis of clinical information and treatment considerations, remains limited in both stability and agreement with expert consensus and that these properties would need to be established before such a component could be relied upon.

### Limitations

This study has several limitations that should be considered when interpreting the findings. First, the analysis was conducted using cases from a single-center MDT conference, which may limit the generalizability of the results to other institutions with different clinical practices or MDT structures. Second, the clinical information provided to the LLMs consisted of structured textual summaries rather than raw clinical data. In real-world clinical environments, decision-making may also involve direct interpretation of imaging studies, pathology slides, and other multimodal data that were not incorporated in the present study. Third, the number of analyzed cases was relatively limited, and larger datasets may be necessary to further validate the observed patterns of concordance and discordance. Fourth, the performance characteristics of LLMs evolve rapidly over time as new model versions are released; therefore, the results of this study should be interpreted within the context of the specific model versions evaluated. In addition, the models were queried in separate blocks over a 7-month period rather than concurrently, and each was evaluated in the version and interface configuration current at that time. Because consumer interfaces do not expose decoding parameters and, for GPT-5.2, route queries between variants without user control, exact reproduction of these outputs is not possible; this constrains reproducibility but reflects the conditions under which clinicians would actually access these models. Finally, concordance estimates depended on how the reference response was defined among repeated queries. We report both the first and the modal response, but neither fully resolves which output should be regarded as a model’s recommendation, and defining this reference remains an open methodological problem in the evaluation of generative models. Complete discordance was defined using the first response of each model and was not recalculated under the modal definition. The multivariate analysis was based on 17 events and is exploratory; the estimate for low MDT agreement derives from 7 cases and should be interpreted with caution.

### Conclusions

Our study demonstrates that LLMs can generate treatment recommendations that show a meaningful degree of alignment with MDT-based decision-making in HPB oncology. Importantly, response stability varied substantially across models, with discordance rates ranging from 12.8% to 30.2%, indicating that concordance alone is insufficient for evaluating LLMs as clinical decision support tools. Although variability in model responses and discordance in certain clinical contexts were observed, these findings suggest that LLM-based systems may feasibly support certain aspects of multidisciplinary clinical reasoning. Before LLMs are integrated into clinical workflows, their response stability must be systematically characterized alongside concordance. As AI technologies continue to evolve, further research integrating multimodal clinical data and multidisciplinary expertise will be essential to better define the role of LLMs within real-world oncologic decision-making processes.

## Supplementary material

10.2196/101137 Multimedia Appendix 1 Treatment category mapping rules and institutional protocols.

10.2196/101137 Multimedia Appendix 2 Annotated model rationales for 4 representative cases.

## Abbreviations

HPB: hepatopancreatobiliary
LLM: large language model
MDT: multidisciplinary team
OR: odds ratio
