# Supplementary material for: Large Language Models in Multidisciplinary Decision-Making for Hepatopancreatobiliary Oncology: Retrospective Comparative Feasibility Study
Source: J Med Internet Res. 2026 Sep 17;28:e101137. doi: 10.2196/101137 (PMC13585308; doi:10.2196/101137)
Supplement: Supplementary file 1 [file jmir-v28-e101137-s001.docx]

**Mapping of multidisciplinary team recommendations to treatment categories**

**1. Purpose**

Multidisciplinary team (MDT) recommendations in hepatopancreatobiliary oncology frequently comprise sequential or combined modalities rather than a single treatment. This appendix specifies the rules by which the documented MDT conclusion, and the treatment recommendation generated by each large language model, were assigned to one of the 5 prespecified categories used in the concordance analysis. The same rules were applied to both, so that the comparison was not affected by differences in how a plan was expressed.

**2. Category definitions**

| **Category** | **Definition** |
| --- | --- |
| Surgery | Resection is the next intervention, with no systemic or local therapy planned before it. |
| Chemotherapy | Systemic therapy is the next intervention, whether the stated intent is neoadjuvant, conversion, adjuvant, or palliative. |
| Radiation therapy | Radiotherapy, with or without concurrent radiosensitizing systemic therapy, is the next intervention. |
| Active surveillance | No antineoplastic treatment is initiated; the patient is followed with planned clinical and radiologic reassessment. |
| Further evaluation | No treatment is initiated pending an additional diagnostic step, such as repeat biopsy, additional imaging, or staging laparoscopy. |

**3. Mapping rules for composite and sequential plans**

Rule 1. A sequential plan is assigned to the modality that is initiated first. A recommendation of neoadjuvant chemotherapy followed by reassessment for resection is therefore categorized as chemotherapy, not as surgery, because chemotherapy is the intervention the patient receives next. This applies irrespective of whether resection is anticipated.

Rule 2. Concurrent modalities are assigned to the dominant local modality. Chemoradiotherapy is categorized as radiation therapy when radiotherapy is the defining component of the plan.

Rule 3. A plan conditional on the result of a pending diagnostic step is categorized as further evaluation, since no treatment is initiated until that result is available.

Rule 4. Perioperative supportive procedures, such as biliary drainage or portal vein embolization, do not constitute a treatment category and were disregarded when the definitive plan was otherwise specified.

Rule 5. The category records the modality only. Regimen, sequencing detail, and intent are not represented in the categorical variable and were therefore not evaluated for concordance.

**4. Institutional treatment protocols**

Management during the study period followed contemporaneous NCCN guidance, with the exception of biliary tract cancer, for which the institutional protocols below were in effect. These are provided so that readers can interpret which underlying plans map to the chemotherapy category.

**Biliary tract cancer**

| **Clinical situation** | **Institutional plan** |
| --- | --- |
| Borderline resectable, or suspected regional nodal metastasis | Neoadjuvant gemcitabine, cisplatin, and nab-paclitaxel, with reassessment for resection |
| Unresectable or metastatic | Palliative gemcitabine, cisplatin, and durvalumab |

**Pancreatic cancer**

| **Clinical situation** | **Institutional plan** |
| --- | --- |
| Borderline resectable | Neoadjuvant FOLFIRINOX, with reassessment for resection |
| Anatomically resectable, but with suspected nodal metastasis or an anatomic configuration in which a margin-negative resection is unlikely, such as uncinate process involvement | Neoadjuvant systemic therapy, with reassessment for resection |
| All other situations | Per contemporaneous NCCN guidance |

Under Rule 1, every situation listed above in which systemic therapy precedes reassessment is categorized as chemotherapy at the time of the MDT conference, including those in which resection is the intended endpoint.

**5. Effect of the simplification**

This categorization records the next treatment modality and not the full therapeutic strategy. Two consequences follow. First, a model recommendation of neoadjuvant chemotherapy followed by surgery and an MDT decision of upfront surgery are counted as discordant, even though both plans anticipate resection; the disagreement concerns the timing of resection rather than whether it is undertaken. Second, agreement within the chemotherapy category does not imply agreement on regimen or on treatment intent, since these are not represented in the variable. Concordance estimates reported in the main text should be read as agreement on the immediate treatment modality.
