# Supplementary material for: Large Language Models in Multidisciplinary Decision-Making for Hepatopancreatobiliary Oncology: Retrospective Comparative Feasibility Study
Source: J Med Internet Res. 2026 Sep 17;28:e101137. doi: 10.2196/101137 (PMC13585308; doi:10.2196/101137)
Supplement: Supplementary file 2 [file jmir-v28-e101137-s002.docx]

**Representative model rationales for discordant and concordant treatment recommendations**

# **1. Purpose and scope**

This appendix presents the free-text rationales generated by the large language model (LLM) for four representative cases, together with an annotation of the reasoning that led to disagreement or agreement with the multidisciplinary team (MDT) decision. It supports the analysis of misclassification direction and content reported in the main text.

Cases were selected to cover both directions of error identified in the quantitative analysis: one recommendation more definitive than the MDT decision, one less definitive, and two concordant recommendations for comparison. They are illustrative and do not substitute for the aggregate concordance, class-wise F1, and error-direction figures reported in Tables 2 to 4 of the main manuscript.

# **2. Case summary**

| **Case** | **Diagnosis and staging** | **Key discriminating input** | **MDT decision** | **Model recommendation** | **Agreement** |
| --- | --- | --- | --- | --- | --- |
| A | Perihilar cholangiocarcinoma, Bismuth type IIIA; adenocarcinoma, moderately differentiated (ERCP biopsy) | CT and MRI disagreed on major vascular invasion; hypotrophy of left lateral hepatic section on CT; no nodal or distant metastasis on PET; total bilirubin 1.28 mg/dL, CA 19-9 45.0 U/mL | Chemotherapy | Surgery | Discordant (more definitive) |
| B | Pancreatic groove or head adenocarcinoma with duodenal invasion, moderately differentiated (endoscopic biopsy) | Partial abutment of first jejunal tributary of SMV only, with no other major vascular invasion; regional nodes in porta hepatis and portocaval space; obstructive jaundice with total bilirubin 6.83 mg/dL; CA 19-9 1566 U/mL, CEA 13.2 ng/mL | Surgery | Chemotherapy (neoadjuvant) | Discordant (less definitive) |
| C | Pancreatic head ductal adenocarcinoma, moderately differentiated (EUS-FNB); borderline resectable | Gastroduodenal artery encasement with main portal vein abutment; small to borderline peripancreatic nodes; CA 19-9 531 U/mL; no thoracic metastasis | Chemotherapy | Chemotherapy (neoadjuvant) | Concordant |
| D | Perihilar cholangiocarcinoma, Bismuth type IV or IIIA; adenocarcinoma, moderately differentiated (biopsy) | Invasion of replaced right hepatic artery and left lobar portal vein; hypermetabolic left supraclavicular, para-aortic, aortocaval and portocaval nodes on PET; 15-mm part-solid right lower lobe nodule; total bilirubin 3.34 mg/dL, AST/ALT 367/507 IU/L | Chemotherapy | Chemotherapy | Concordant |

ALT: alanine aminotransferase; AST: aspartate aminotransferase; CA 19-9: carbohydrate antigen 19-9; CEA: carcinoembryonic antigen; CT: computed tomography; ERCP: endoscopic retrograde cholangiopancreatography; EUS-FNB: endoscopic ultrasound-guided fine-needle biopsy; MRI: magnetic resonance imaging; PET: positron emission tomography; SMV: superior mesenteric vein.

# **3. Discordant recommendations**

## **3.1 Case A — recommendation more definitive than the MDT decision**

### ***Model rationale***

*Confirmed perihilar cholangiocarcinoma of Bismuth type IIIA, with an approximately 4-cm infiltrative tumor on MRI. CT raised the possibility of invasion of major vessels including the portal vein and hepatic artery, but MRI showed no definite evidence of major vascular invasion. PET showed no nodal or distant metastasis, and hepatic function is adequately preserved despite the patient being 72 years of age; curative-intent resection including right hepatectomy is therefore feasible.*

### ***Annotation***

**Unreconciled discordance between imaging modalities.** CT described the tumor as abutting both main portal venous branches and the right hepatic artery, probably with tumor invasion, whereas MRI reported no definite evidence of major vascular invasion. The model adopted the more permissive reading without acknowledging the conflict or reflecting the residual uncertainty in its recommendation. The MDT treated the same discrepancy as an argument against proceeding directly to resection.

**Omission of a determinant of surgical feasibility.** CT reported hypotrophy of the left lateral hepatic section. In Bismuth type IIIA disease the planned procedure is a right-sided hepatectomy, for which the left liver constitutes the future liver remnant; hypotrophy of that segment bears directly on remnant adequacy and on the need for preoperative volume optimization. The model cited preserved hepatic function in support of resection but did not address remnant volume, which is a distinct consideration.

## **3.2 Case B — recommendation less definitive than the MDT decision**

### ***Model rationale***

*Diagnosed as carcinoma of the pancreatic groove region, with invasion extending into the duodenum (second and third portions). On imaging the tumor lies adjacent to a branch of the superior mesenteric vein, and above all CA 19-9 is markedly elevated at 1566 U/mL, with CEA (13.2) and bilirubin (6.83) also considerably raised. Given the patient’s young age of 54 years and the high tumor markers, a strategy of neoadjuvant chemotherapy to control systemic disease and downstage the tumor is required rather than immediate surgery.*

### ***Annotation***

**Anatomic features that do not affect resectability treated as adverse.** Duodenal invasion was cited in support of deferring surgery, although the duodenum is removed en bloc at pancreaticoduodenectomy and its involvement does not alter resectability. The only reported venous contact was partial abutment of a first jejunal tributary of the superior mesenteric vein, with no other evidence of major vascular invasion, and the involved nodes lay within the standard lymphadenectomy field. By anatomic criteria the disease was resectable, as the MDT concluded.

**Tumor marker interpreted without accounting for biliary obstruction.** A CA 19-9 of 1566 U/mL was presented as the principal evidence of aggressive biology in a patient with a total bilirubin of 6.83 mg/dL. Obstructive jaundice raises CA 19-9 independently of tumor burden, and the model neither acknowledged this confounding nor considered reassessment after biliary drainage. The same reasoning also treated an elevated bilirubin as an independent adverse prognostic feature rather than as a consequence of the biliary obstruction produced by the tumor.

**Nonspecific use of demographic information.** Age 54 years was cited in support of deferring surgery. Younger age with preserved performance status is not in itself an indication for neoadjuvant therapy in anatomically resectable disease, and the direction of the inference was not explained.

**Interpretive caveat.** Neoadjuvant therapy for anatomically resectable pancreatic ductal adenocarcinoma remains an area of legitimate clinical debate, so the recommended category is not indefensible in isolation. The concern illustrated here is that the stated justification rested on features that either do not bear on resectability or were confounded by biliary obstruction, rather than on the trial evidence that would support a neoadjuvant approach.

## **3.3 Direction of error**

Cases A and B illustrate the two directions identified in the quantitative analysis. Of 153 discordant first responses across the 4 models, 97 recommended a less definitive strategy than the MDT decision and 56 recommended a more definitive one. The most frequent single transitions were from an MDT decision of chemotherapy to a model recommendation of surgery (52), from surgery to chemotherapy (41), and from chemotherapy to further evaluation (40); among the 25 cases in which the MDT recommended surgery, the models recommended chemotherapy instead in 8 to 13 cases, depending on the model.

Case B corresponds to the second of these transitions and Case A to the first. Notably, the models did not under-recommend surgery in aggregate: surgery was recommended in 20 to 29 cases against 25 MDT surgical decisions. Misclassification therefore concerned which patients were assigned to surgery rather than how often surgery was recommended, which is consistent with the consistently lower class-wise F1 for surgery than for chemotherapy reported in the main text. The two cases below are matched in that respect: both involve a surgical decision boundary, and the model erred in opposite directions across it.

# **4. Concordant recommendations**

## **4.1 Case C**

### ***Model rationale***

*Confirmed ductal adenocarcinoma of the pancreatic head. On imaging the tumor encases the gastroduodenal artery and abuts the main portal vein, corresponding to borderline resectable disease. CA 19-9 is elevated at 531 U/mL, so a strategy of neoadjuvant chemotherapy to downstage the tumor and increase the likelihood of complete resection is preferred.*

### ***Annotation***

The model applied standard anatomic resectability criteria to the reported arterial and venous relationships, reached the correct borderline resectable classification, and additionally invoked the elevated CA 19-9 as a marker of biologic risk. Both the classification and the treatment sequence matched the MDT decision. Notably, the same tumor marker reasoning that was appropriate here was misapplied in Case B, where the value was confounded by obstructive jaundice and the anatomy was resectable.

## **4.2 Case D**

### ***Model rationale***

*Perihilar cholangiocarcinoma of Bismuth type IV or IIIA, invading the right hepatic artery arising from the superior mesenteric artery (an anatomic variant) and the left lobar portal vein. PET in particular shows findings suspicious for widespread distant nodal metastasis, including the left supraclavicular region (SUV 5.2), para-aortic and aortocaval areas. Chest CT additionally demonstrates a nodule that may represent pulmonary metastasis. Systemic chemotherapy for disease control is therefore more appropriate than surgery.*

### ***Annotation***

The model integrated findings across three modalities — cross-sectional imaging for local vascular involvement, PET for nodal disease, and chest CT for the indeterminate pulmonary nodule — and correctly distinguished nodal disease beyond the surgical field from regional nodal disease. The recommendation and its stated basis both matched the MDT decision.

# **5. Summary of observed reasoning patterns**

Across the four cases, discordance did not arise from misclassification of the primary diagnosis, which was correct in every instance, nor from failure to identify the reported imaging findings. It arose instead from three recurring patterns: incomplete reconciliation of conflicting reports between imaging modalities (Case A); omission of feasibility constraints that were central to the MDT deliberation, such as future liver remnant adequacy (Case A); and misweighting of individual findings, in which features that do not bear on resectability or that are confounded by an intercurrent condition were treated as decisive (Case B). In the concordant cases the same categories of information were weighted in a manner consistent with the reasoning documented at the conference. Because the primary diagnosis and the reported findings were identified correctly throughout, these appear to be failures of clinical weighting rather than of information retrieval.

# **6. Notes on preparation**

All direct and indirect identifiers were removed before analysis; cases are indexed A to D and no dates, names, medical record numbers, or institutional identifiers are reproduced.

Model rationales were generated in Korean and have been translated into English for this appendix. Translation preserved the content and structure of the original output; no clinical claim was added, removed, or qualified.

Model: Gemini 3 Pro. The model version was pinned in the model selector for every query rather than relying on the application default, so all iterations were served by the same version. Queries were performed after the close of the MDT enrollment period, and the MDT decision was not included in the model input. For each of the four cases the recommended treatment category was identical across all four query iterations, so the case classification shown above applies equally to the first response used in the quantitative analysis reported in the main text; the rationale text reproduced above is taken from the fourth iteration.

Abbreviations: CT: computed tomography; LLM: large language model; MDT: multidisciplinary team; PET: positron emission tomography; SUV: standardized uptake value.
